# Measuring mathematics self-efficacy: Multitrait-multimethod comparison

**DOI:** 10.3389/fpsyg.2023.1108536

**Published:** 2023-03-07

**Authors:** Wenhua Yu, Shuodi Zhou, Yu Zhou

**Affiliations:** ^1^School of Mathematics and Statistics, Shandong Normal University, Jinan, China; ^2^Department of Mathematics, London School of Economics and Political Science, London, United Kingdom

**Keywords:** self-efficacy, mathematics self-efficacy, multitrait-multimethod design, confirmatory factor analysis, academic performance in mathematics

## Abstract

Previous studies had shown that there is a certain relationship between mathematics self-efficacy and math performance. For students, parents, and front-line scholars, it is urgent and important to study the measurement relationship between math achievement and self-efficacy. The research aimed to observe how to measure mathematics self-efficacy and find which of the three traits and which of the three methods better reflect individuals’ self-efficacy. The present study used a multitrait-multimethod (MTMM) design to measure mathematics self-efficacy by constructing the confirmatory factor analysis (CFA) model. “Number and Algebra,” “Graphics and Geometry,” and “Synthesis and Practice” were considered three traits, and General-Math-Task-referenced self-efficacy, Unconventional-Math-Problem-referenced self-efficacy, and Motivated Strategies for Learning Questionnaire (MSLQ) self-efficacy were discussed as three methods to study. A questionnaire survey was used to obtain data. A total of 100 students completed all the questionnaires. Excel was used to collect math scores, and SPSS version 26.0 and AMOS version 26.0 were used to manage the data, confirm a hypothesis, and build a model by using MTMM design and CFA. CFA was used to verify convergent validity and discriminant validity. A total of eight models were constructed in the study that includes first-order CFA models and second-order CFA models, and model D was finally selected as the most perfect model in the second-order CFA model. The results showed that the “Synthesis and Practice” fields were the most significant reflection of self-efficacy among the three traits. MSLQ was the most significant reflection of self-efficacy among the three methods. It is beneficial to improve the level of self-efficacy from the aspect of mathematics subject. In addition, the research confirmed that CFA can support MTMM data for data modeling and found that the correlation between the Unconventional-Math-Problem-referenced self-efficacy and MSLQ is higher than that of General-Math-Task-referenced self-efficacy in the second-order model. It makes certain theoretical significance for improving students’ mathematics self-efficacy levels.

## 1. Introduction

Bandura’s social cognitive theory (Bandura, 1977) defined self-efficacy as people’s view on how well he or she performs at specific assignments to reach a given level of performance. This shows how individuals, behaviors, and environmental aspects can influence one’s actions. In general, the better self-efficacy the students have, the higher targets they aim for, the harder quests they seek, and the more unyielding with obstacles (Bandura, 1986). People tend to do the things they trust they can do well and resist those in that they do not have confidence in (Bandura, 1997). Therefore, how students view themselves has a great effect on their actions, and their belief that they can perform well can encourage them to make an effort at studying. Previous studies have suggested that the higher the self-efficacy, the more motivated and the better the academic performance of students (Pajares and Valiante, 1997). In the discipline of mathematics, mathematics self-efficacy refers to the evaluation of one’s confidence to succeed in a math problem (Hackett and Betz, 1989). The Mathematics Self-Efficacy Scale was created by Betz and Hackett (1983). Theoretically, it is believed that individual mathematics self-efficacy can affect mathematics performance by influencing behavior and psychological process (Bandura, 1986; Bandura, 1997).

In empirical research, it is found that mathematics self-efficacy can accurately predict how well the outcomes are, by exploring the effect of mental ability and self-efficacy on math performance (Pajares and Kranzler, 1995). Mathematics self-efficacy uses both scores from lessons and exams to predict performance in math (Grigg et al., 2018). In math problem-solving, Pajares and Miller (1994) verified the function of self-efficacy in predicting and mediating. The results showed that self-efficacy is actually better at predicting their performance in solving problems than the other methods used to predict it. For measuring individuals’ self-efficacy, Pajares and Miller (1995) proposed that this measure has to be strongly linked to the assignment being done; furthermore, it should show their confidence in succeeding at math problems and courses. According to Bandura (1997), as a key internal factor, self-efficacy refers to one’s own view of his or her capability at performing tasks. Linnenbrink and Pintrich (2003) suggested that mathematics self-efficacy means opinions of abilities that can be expressed as “I think I can subtract numbers below 100”. Animasaun and Abegunrin (2017) proposed that students with a low and medium level of mathematics self-efficacy may be subjected to either enactive mastery experiences (actual performances) or verbal persuasion in order to boost their self-efficacy. There exists a wide gap in home support, self-efficacy, active learning strategies, the attitude of students toward mathematics, and academic achievement between students with a weak, average, and strong interest in mathematics (Animasaun, 2021).

In measuring disciplinary self-efficacy, qualitative and quantitative methods were usually used at the same time. Dalgety and Coll (2006) used qualitative and quantitative data to observe changes in students’ self-efficacy in the chemistry field. Webb-Williams (2018) investigated children’s self-efficacy in science. Gao (2020) used a semi-structured interview using a “Q-sorting” process to investigate where mathematical self-efficacy came from in-depth. In contrast, some studies use quantitative methods only to measure disciplinary self-efficacy. Ding et al. (2022) tested the measurement invariance of mathematics self-concept and self-efficacy in the Programme for International Student Assessment (PISA) using multiple-group confirmatory factor analysis (CFA) and the alignment method. Through exploratory and confirmatory analyses and pilot testing, Luo et al. (2021) measured the self-efficacy of students in activities in STEM. Larsen and Jang (2021) used factor score path analysis on multiple levels to measure the relationship among mathematical achievement, instructional practices, and self-efficacy of students. Wong et al. (2021) determined the relationship between academic hardiness in science, learning science theory, and self-efficacy of students in studying science in Malaysian secondary schools, using the technology of modeling structural equations. In addition, Liu et al. (2020) described a link among task-specific and domain self-efficacy and math problem posing in detail using linear regression, generalized additive, and piecewise regression models. All these studies provide some references for the study of discipline self-efficacy.

Multitrait-multimethod (MTMM) modeling was shown as effective during the last 50 years of studies on psychology (Byrne, 2006). MTMM uses at least two methodologies to experiment with at least two traits. MTMM matrix can evaluate the effectiveness of convergence and divergence (Byrne, 2012; Kline, 2015). An effective and nice way to test the validity of a construct with MTMM is CFA in the structural equation model (SEM; Tildesley et al., 1995). Bong and Hocevar (2002) provided good use of MTMM in measuring self-efficacy. In their MTMM design, math, Korean, and English were discussed as three traits; Problem-referenced self-efficacy, Task-referenced self-efficacy, and Motivated Strategies for Learning Questionnaire (MSLQ) self-efficacy were considered three methods to measure self-efficacy. It is confirmed that factor analytic procedures of high order are useful to analyze the validity problems of convergent and discriminant.

How to calculate self-efficacy in math, especially using MTMM, which is so efficient and elegant as mentioned above? This study used an MTMM design to measure mathematics self-efficacy. “Number and Algebra,” “Graphics and Geometry,” and “Synthesis and Practice” were considered three traits, and General-Math-Task-referenced self-efficacy, Unconventional-Math-Problem-referenced self-efficacy, and MSLQ self-efficacy were discussed to be three methods. MSLQ self-efficacy was used to measure the cognitive level of academic events in the three fields and help students recognize the level of self-efficacy of learning motivation. This research aimed to explore how to measure mathematics self-efficacy and to find whether MTMM is a significant measurement of students’ mathematics self-efficacy. The following studies were determined (1) to verify whether the MTMM can measure students’ mathematics self-efficacy by establishing a high-order CFA model, (2) to find which of the three traits has more significant effects on students’ mathematics self-efficacy through MTMM, and (3) to find which of the three methods has more significant effects on students’ self-efficacy through MTMM.

## 2. Research methodology

### 2.1. Materials and methods

#### 2.1.1. Participants and procedure

Due to the COVID-19 pandemic, the participants were only limited to two schools in Kaifeng and Shangqiu, Henan Province, China. Two rounds of tests were conducted and students of the autumn term in year 7 were selected as the research objects. In the round of prediction, 70 students were selected to test the quality of the test tools. The test tools were revised to have better validity according to the test results and expert opinions. During formal testing, 72 students from Kaifeng and 70 students from Shangqiu were selected for the offline test. General-Math-Task-referenced self-efficacy questionnaire, Unconventional-Math-Problem-referenced self-efficacy questionnaire, and MSLQ self-efficacy were distributed to teachers, who handed them out to students in the form of homework. Table 1 shows the number of questionnaires. Materials were collected online and offline, and 100 valid questionnaires were finally obtained for data analysis. The studies involving human participants were reviewed and approved by the Ethics Committee of Shandong Normal University. The participants provided their written informed consent to participate in this study.

**Table 1:** Numbers of questionnaire questions.

Methods	General-Math-Task-referenced self-efficacy questionnaire	Unconventional-Math-Problem-referenced self-efficacy questionnaire	Motivated Strategies for Learning Questionnaire	Total numbers of questions
Traits				
Number and Algebra	3	3	6	12
Graphics and Geometry	3	3	6	12
Synthesis and Practice	3	3	6	18
Total numbers of questions	9	9	18	36

### 2.2. Measurements

#### 2.2.1. General-math-task-referenced self-efficacy questionnaire

In the “Mathematics Curriculum Standards for Compulsory Education (2011 Edition) (Ministry of Education of China, 2011),” mathematics learning was divided into four areas, namely, “Number and Algebra,” “Graphics and Geometry,” “Probability and Statistics,” and “Synthesis and Practice.” Among them, Synthesis and Practice is a comprehensive subject field based on the first three fields. Considering “Probability and Statistics” is much simpler than the others in junior high school, this study removed this field. Therefore, in this study, “Number and Algebra,” “Graphics and Geometry,” and “Synthesis and Practice” were included as three traits in the General-Math-Task-referenced self-efficacy questionnaire. For each trait, three questions were selected from relevant mathematical exercise questions in the seventh grade in the PEP edition, whose difficulty involves the following three levels: easy, medium, and difficult. For each question, different scores of self-efficacy were given due to different degrees of difficulty in question. For example, the second question of General-Math-Task-referenced self-efficacy in “Number and Algebra” is 5 marks in full. Finally, the experimenter asked the students to answer how confident he/she is to do this question on a scale of 0 to 5. The full score of self-efficacy for each question is shown in Table 2. This is the common process in verifying the belief of self-efficacy with given problems (Bandura, 1997).

**Table 2:** Scores of each index of the questionnaires.

Methods	General-Math-Task-referenced self-efficacy questionnaire				Unconventional-Math-Problem-referenced self-efficacy questionnaire				Motivated Strategies for Learning Questionnaire							Total scores
Self-efficacy total score of each item	1	2	3	Total scores	1	2	3	Total scores	1	2	3	4	5	6	Total scores	
Traits																
Number and Algebra	5	5	5	15	7	6	7	20	7	7	7	7	7	7	42	77
Graphics and Geometry	6	8	6	20	7	8	9	24	7	7	7	7	7	7	42	86
Synthesis and Practice	10	9	6	25	9	8	9	26	7	7	7	7	7	7	42	93
Total scores				60				70							126	256

The columns represent the traits, the methods represented by the rows, the number of questions indicated by the numbers in the first row, and the numbers in the following rows indicate the scores and total scores of the methods in a certain trait.

#### 2.2.2. Unconventional-math-Problem-referenced self-efficacy questionnaire

In light of the three traits, a total of 10 Unconventional-Math-Problem-referenced questions were selected that were similar to the cognitive level of seventh-grade students and had less correlation with the in-class learning content. According to the pre-test results, one question was deleted and three questions were kept in each trait. The Unconventional-Math-Problem-referenced self-efficacy questions are mostly open-ended questions that involve asking students to pose different levels of math problems or to pose and solve problems. Similar to the General-Math-Task-referenced self-efficacy questionnaire, different questions were given different total self-efficacy scores. For example, question 2 of Unconventional-Math-Problem-referenced self-efficacy in Graphics and Geometry is 8 marks in full. Then, the students assessed the degree of confidence in their solutions.

#### 2.2.3. Motivated strategies for learning questionnaire self-efficacy

Bong and Hocevar (2002) introduced MSLQ self-efficacy, and it is used to measure self-efficacy, which is dedicated to measuring a certain field of mathematics subjects, and is a measure of the degree of recognition of statements of general academic events. It consists of the following six items: “I’m certain that I can understand what is taught in (a specific school subject) class,” “I expect to do very well in (a subject) class,” “I am sure that I can do an excellent job on the problems and tasks assigned for (a subject) class,” “I know that I will be able to learn the material for (a subject) class,” “My study skills are excellent in (a subject) class,” and “I think I will receive a good grade in (subject) class.” In light of “Number and Algebra,” “Graphics and Geometry,” and “Synthesis and Practice” in this article, 6 items were measured, respectively. Therefore, the questionnaire includes a total of 18 items. Response categories ranged from the 7-point Likert scale. The scores are shown in Table 2.

#### 2.2.4. Numbers and scores of questionnaire questions

Details are summarized in Tables 1, 2.

### 2.3. Data analysis

Excel and SPSS version 26.0 were used for data statistics and collation, which aimed to analyze the Cronbach coefficient of each item and each facet, and “1” represents girls and “2” represents boys. Then, SPSS version 26.0 and Amos version 26.0 were used to analyze if these statistics were reliable and valid and calculate their model fit indexes. Amos was used to draw a model diagram based on data collected by SPSS, in which three traits and three methods affect each other. The final model diagram is shown in the following figure, in which reliability and validity, model fit, etc., were output.

## 3. Analysis and discussion of results

### 3.1. Descriptive statistics of the model

According to the value of descriptive statistics, it was found that the values were almost significant of each trait, each method, and each item in the model. The table shows the reliability of the Cronbach coefficient, composite reliability, and convergent validity to test if the item and model are reliable and valid.

Table 3 shows indicators of factor loadings. Hair et al. (2009) deemed that loading estimates have to be more than 0.5, even more than 0.7 after standardizing. About the explanation of each factor loading of each trait in this article, for example, the factor loading of Unconventional-Math-Problem-referenced self-efficacy in Number and Algebra is 0.427, which represented the index of self-efficacy of Unconventional-Math-Problem-referenced self-efficacy in Number and Algebra is 0.427. That is to say, the index of self-efficacy of Unconventional-Math-Problem-referenced self-efficacy in Number and Algebra is increased by 1, and the value of Number and Algebra can increase by 0.427 in the whole model. The rest are the same as earlier. Through comparison, it can be found that the factor loadings of the three methods were basically acceptable, and the data were as follows: in Number and Algebra (Unconventional-Math-Problem-referenced self-efficacy = 0.427, General-Math-Task-referenced self-efficacy = 0.160, and MSLQ = 0.582), in Graphics and Geometry (Unconventional-Math-Problem-referenced self-efficacy = 0.581, General-Math-Task-referenced self-efficacy = 0.326, and MSLQ = 0.686), and in Synthesis and Practice (Unconventional-Math-Problem-referenced self-efficacy = 0.612, General-Math-Task-referenced self-efficacy = 0.398, and MSLQ = 0.707). Among them, “Synthesis and Practice” was the most important part of the three traits. Most of the factor loadings of the three traits were ideal in Unconventional-Math-Problem-referenced self-efficacy (Graphics and Geometry = 1.052, Number and Algebra = 0.895, and Synthesis and Practice = 1.186), in General-Math-Task-referenced self-efficacy (Graphics and Geometry = 0.640, Number and Algebra = 0.417, and Synthesis and Practice = 0.835), and in MSLQ (Graphics and Geometry = 0.954, Number and Algebra = 0.949, and Synthesis and Practice = 0.815). It was found that MSLQ has the greatest impact among the three methods.

**Table 3:** Descriptive statistics of the model.

Facets	Questions	Unstd	S.E.	*T*-value	*p*	Std	*α*	CR	AVE
Alge	Pro	1				0.427	0.301	0.358	0.182
	Task	0.308	0.235	1.307	0.191	0.160			
	MSLQ	1.515	0.415	3.652	***	0.582			
Geo	Pro	1				0.581	0.457	0.549	0.305
	Task	0.088	0.112	0.784	0.433	0.326			
	MSLQ	1.466	0.428	3.421	***	0.686			
Prac	Pro	1				0.612	0.551	0.600	0.344
	Task	0.754	0.297	2.540	0.011	0.398			
	MSLQ	0.765	0.233	3.283	0.001	0.707			
Pro	Geo	1				1.052	0.821	1.033	1.105
	Alge	1.249	0.280	4.461	***	0.895			
	Prac	1.638	0.419	3.905	***	1.186			
Task	Geo	1				0.640	0.574	0.676	0.427
	Alge	4.589	5.085	0.902	0.367	0.417			
	Prac	14.406	15.044	0.958	0.338	0.835			
MSLQ	Geo	1				0.954	0.877	0.934	0.825
	Alge	1.421	0.226	6.275	***	0.949			
	Prac	0.874	0.173	5.047	***	0.815			
	AP1	1				0.691	0.607	0.638	0.376
Alge_Prob	AP2	0.321	0.107	3.007	0.003	0.460			
	AP3	0.944	0.317	2.975	0.003	0.663			
	AT1	1				0.407	0.432	0.536	0.348
Alge_Task	AT2	0.312	0.207	1.513	0.130	0.175			
	AT3	1.723	2.259	0.763	0.445	0.921			
	AQ1	1				0.860	0.884	0.885	0.565
	AQ2	0.636	0.100	6.378	***	0.602			
Alge_MSLQ	AQ3	0.789	0.097	8.172	***	0.726			
	AQ4	0.866	0.098	8.873	***	0.769			
	AQ5	0.788	0.100	7.871	***	0.707			
	AQ6	0.968	0.100	9.692	***	0.817			
	GP1	1				0.415	0.605	0.622	0.366
Geo_Prob	GP2	2.859	1.107	2.583	0.010	0.739			
	GP3	1.983	0.684	2.898	0.004	0.616			
	GT1	1				0.089	0.559	0.641	0.453
Geo_Task	GT2	20.821	33.959	0.613	0.540	0.937			
	GT3	10.88	13.051	0.834	0.404	0.687			
	GL1	1				0.692	0.826	0.827	0.450
	GL2	0.578	0.141	4.109	***	0.460			
Geo_MSLQ	GL3	1.020	0.171	5.957	***	0.689			
	GL4	1.039	0.175	5.943	***	0.687			
	GL5	0.969	0.172	5.624	***	0.645			
	GL6	1.232	0.184	6.698	***	0.803			
	PR1	1				0.432	0.430	0.599	0.406
Prac_Prob	PR2	1.541	1.566	0.984	0.325	0.994			
	PR3	0.533	0.279	1.912	0.056	0.207			
	PT1	1				0.503	0.721	0.786	0.576
Prac_Task	PT2	1.996	0.557	3.581	***	1.050			
	PT3	0.716	0.148	4.842	***	0.609			
	PS1	1				0.654	0.829	0.834	0.465
	PS2	0.632	0.168	3.760	***	0.424			
Prac_MSLQ	PS3	1.154	0.200	5.775	***	0.694			
	PS4	1.22	0.190	6.436	***	0.806			
	PS5	1.02	0.180	5.652	***	0.675			
	PS6	1.299	0.208	6.245	***	0.769			

Alge, Number and Algebra; Geo, Graphics and Geometry; Prac, Synthesis and Practice; Pro, Unconventional-Math-Problem-referenced self-efficacy; Task, General-Math-Task-referenced self-efficacy; MSLQ, Motivated Strategies for Learning Questionnaire; Alge_Prob, Number and Algebra questions in Unconventional-Math-Problem-referenced self-efficacy questionnaire; Geo_Prob, Graphics and Geometry questions in Unconventional-Math-Problem-referenced self-efficacy questionnaire; Prac_Prob, Synthesis and Practice questions in Unconventional-Math-Problem-referenced self-efficacy questionnaire; ***p < 0.001.

Table 3 shows the factor loading of Graphics and Geometry, Number and Algebra, and Synthesis and Practice in the Unconventional-Math-Problem-referenced self-efficacy, General-Math-Task-referenced self-efficacy, and MSLQ. The factor loadings of Unconventional-Math-Problem-referenced self-efficacy in Number and Algebra were 0.691, 0.460, and 0.663, which indicated that they were basically acceptable. The factor loadings of General-Math-Task-referenced self-efficacy in Number and Algebra were 0.407, 0.175, and 0.921, which were basically acceptable. The factor loadings of MSLQ in Number and Algebra were 0.860, 0.602, 0.726, 0.769, 0.707, and 0.817, which were basically acceptable. The factor loadings of Unconventional-Math-Problem-referenced self-efficacy in Graphics and Geometry were 0.415, 0.739, and 0.616, which were mostly acceptable. The factor loadings of General-Math-Task-referenced self-efficacy in Graphics and Geometry were 0.089, 0.937, and 0.687, which were mostly acceptable. The factor loadings of MSLQ in Graphics and Geometry were 0.692, 0.460, 0.689, 0.687, 0.645, and 0.803, which were mostly acceptable. The factor loadings of Unconventional-Math-Problem-referenced self-efficacy in Synthesis and Practice were 0.432, 0.994, and 0.207, which indicated that they were basically acceptable. The factor loadings of General-Math-Task-referenced self-efficacy in Synthesis and Practice were 0.503, 1.050, and 0.609, which were within the acceptable range. The factor loadings of MSLQ in Synthesis and Practice were 0.654, 0.424, 0.694, 0.806, 0.675, and 0.769, which were mostly acceptable.

Table 3 presents the reliability and validity of the questionnaire. For reliability, two methods of Cronbach coefficient and composite reliability were used. The table shows that Cronbach coefficients of the Unconventional-Math-Problem-referenced self-efficacy questionnaire, the General-Math-Task-referenced self-efficacy questionnaire, and MSLQ were all close to 0.70, which was introduced by Nunnally and Bernstein (1994).

The table shows how reliable each question, three methods, and three traits are. When talking about the CR value, Hair et al. (2009) thought that the rule of thumb for both calculations of reliability should be at least 0.7, which means reliable, and above 0.6, which is acceptable. Among the three traits, Graphics and Geometry (CR = 0.549) and Synthesis and Practice (CR = 0.600) had higher reliability than others, while Number and Algebra (CR = 0.358) had low reliability. The reliability of MSLQ (CR = 0.934) and General-Math-Task-referenced self-efficacy (CR = 0.676) was acceptable, while the reliability of Unconventional-Math-Problem-referenced self-efficacy (CR = 1.033) was the highest. In Number and Algebra, among the composite reliability of Unconventional-Math-Problem-referenced self-efficacy, General-Math-Task-referenced self-efficacy, and MSLQ, the reliability of Unconventional-Math-Problem-referenced self-efficacy (CR = 0.638) and General-Math-Task-referenced self-efficacy (CR = 0.536) was acceptable, and the reliability of MSLQ (CR = 0.885) was the highest among all questions. In Graphics and Geometry, among the composite reliability of Unconventional-Math-Problem-referenced self-efficacy, General-Math-Task-referenced self-efficacy, and MSLQ, the reliability of Unconventional-Math-Problem-referenced self-efficacy (CR = 0.622) and General-Math-Task-referenced self-efficacy (CR = 0.641) was acceptable, and the reliability of MSLQ (CR = 0.827) was the highest among all questions. In Synthesis and Practice, among the composite reliability of Unconventional-Math-Problem-referenced self-efficacy, General-Math-Task-referenced self-efficacy, and MSLQ, the reliability of Unconventional-Math-Problem-referenced self-efficacy (CR = 0.599) and General-Math-Task-referenced self-efficacy (CR = 0.786) was acceptable, and the reliability of MSLQ (CR = 0.834) was the highest among all questions.

If the average variance extracted (AVE) is at least 0.5, then this is a fitting rule of thumb with suitable convergence (Hair et al., 2009). Table 3 illustrates the convergent validity of three methods and three traits. Among the three traits, the validity of Graphics and Geometry (AVE = 0.305) and Synthesis and Practice (AVE = 0.344) was close to 0.5, and the validity of Number and Algebra (AVE = 0.182) was not high. Among the Unconventional-Math-Problem-referenced self-efficacy, General-Math-Task-referenced self-efficacy, and MSLQ, the validity of Unconventional-Math-Problem-referenced self-efficacy (AVE = 1.105) and MSLQ (AVE = 0.825) was both higher than 0.5, while the validity of General-Math-Task-referenced self-efficacy (AVE = 0.427) was not higher than 0.5. In Number and Algebra, the validity of Unconventional-Math-Problem-referenced self-efficacy (AVE = 0.376), General-Math-Task-referenced self-efficacy (AVE = 0.348), and MSLQ (AVE = 0.565) was close to 0.5. In Graphics and Geometry, the validity of Unconventional-Math-Problem-referenced self-efficacy (AVE = 0.366), General-Math-Task-referenced self-efficacy (AVE = 0.453), and MSLQ (AVE = 0.450) was close to 0.5. In Synthesis and Practice, the validity of Unconventional-Math-Problem-referenced self-efficacy (AVE = 0.406), General-Math-Task-referenced self-efficacy (AVE = 0.576), and MSLQ (AVE = 0.465) was close to 0.5. In general, the convergent validity of each method, each trait, and each item is good.

### 3.2. Goodness-of-fit indexes of the CFA model

Lai et al. (2010) proposed that we can compare the CFA of the first order and the second order by calculating the target coefficient to determine if it fits with statistics. The T value nearer one suggests that the second-order CFA is appropriate to substitute the first-order CFA, and the model is accurate. In this study, the second-order CFA model is appropriate to substitute the first-order CFA model.

This model fit indexes of the first-order CFA model and the higher-order CFA model are shown in Table 4. A total of eight models had been constructed in this study. Models 1 to 4 are the first-order SEM, and models A to D are the second-order SEMs. In models 1 and 2, the correlation of trait and method, respectively, cannot be found, and the fit indexes of the two models are not ideal. Model 3 is the first-order model in that traits and methods are not correlated, which is more acceptable than the model fit indexes of the first two models. Model 4 is the first-order model of 9 correlated methods and traits, which reflected the unique addition of a single method and single trait. Compared with the first three models, the model fit indexes of model 4 are better. Model A is the second-order model with three correlated methods, model B is the second-order model with three correlated traits, and model C is the second-order model with three traits correlated with three methods; the method and traits are not correlated at the same time, standardized root mean square residual (SRMR) value cannot be estimated successfully, and model C had improved compared with the previous models. Model D is the second-order model based on model C except for General-Math-Task-referenced self-efficacy, and the value of SRMR could be estimated. Model D is the preset perfect model, and the model fit indexes were the best of all models.

**Table 4:** Goodness-of-fit indexes of confirmatory factor analysis model.

Model	Description	χ2/df	GFI	AGFI	TLI	CFI	RMSEA	SRMR
1	3 correlated method first-order factors only	1.809	0.645	0.599	0.678	0.698	0.090	0.090
2	3 correlated trait first-order factors only	2.191	0.554	0.497	0.527	0.556	0.110	0.120
3	3 correlated method and 3 correlated trait first-order factors with no trait-method correlation	1.677	0.675	0.608	0.731	0.764	0.083	–
4	9 correlated trait-method combination first-order factors	1.662	0.676	0.613	0.737	0.767	0.082	0.085
A	3 correlated method second-order factors	1.652	0.671	0.623	0.741	0.761	0.081	0.089
B	3 correlated trait second-order factors	1.835	0.650	0.600	0.668	0.694	0.092	0.113
C	3 correlated method and 3 correlated trait second-order factors with no trait-method correlation	1.651	0.673	0.618	0.741	0.766	0.081	–
D	Model C with Task method factor removed	1.645	0.675	0.624	0.744	0.766	0.081	0.090

A theory that a model illustrates reasonable fit when the change in data is less or equal to 3.0 was introduced (Kline, 2004): χ^2^/df ≤ 3. Table 4 shows the value of all models which is less than or equal to 3, indicating that the fit indexes of all of them are reasonable.

In Table 4, the Adjusted Goodness-of-Fit Index (AGFI) and Goodness-of-Fit Index (GFI) figures in all models were between 0.5 and 0.7, close to the value which is generally accepted value of 0.90 (Hooper et al., 2008). With the model changing from second order to first order, it was found that the fit indexes of model D were better.

The comparative fit index (CFI ≥ 0.95) is currently seen as illustrating goodness-of-fit (Hu and Bentler, 1999). CFI is an index of goodness-of-fit. The larger it is the better. It is a number between 0.0 and 1.0. Furthermore, it tries to set the level of freedom in the model straight into the calculation to adjust model complexity or parsimony (Iacobucci, 2009). Marsh et al. (2004) proposed that we should not criticize it when CFI is a bit below 0.95 or SRMR is a bit below 0.09. The CFI values of most models were between 0.7, which was close to the index of goodness-of-fit. With the model changing from the first order to the second order, it was found that model D’s fit index was more fit than previous models.

The issue was tackled using the Non-Normed Fit Index (NNFI, also called Tucker–Lewis index), which prefers models that are simpler. However, when the samples are small, the value of NNFI could suggest bad fitness even though other data show fair fitness. The last issue about NNFI is that its non-normed characteristic figures could be higher than 1.0, therefore, making it difficult to analyze (Hooper et al., 2008). The indicators of each model were close to 0.8. On the whole, model D was closer to the standard indicators.

Root mean square error of approximation (RMSEA) values between 0.05 and 0.10 indicated good fitness, values higher than 0.10 indicated bad fitness, values between 0.08 and 0.10 indicated mediocre fitness, and values lower than 0.08 indicated nice fitness (MacCallum et al., 1996). All models’ indicators in Table 5 were almost 0.08, which means that they were close to medium fit.

**Table 5:** Correlation indexes of first-order confirmatory factor analysis model.

Factor	1	2	3	4	5	6	7	8	9
Alge_Task	**0.590**								
Prac_MSLQ	0.334	**0.682**							
Prac_Task	0.383	0.286	**0.759**						
Prac_Prob	0.379	0.398	0.441	**0.637**					
Alge_MSLQ	0.223	0.768	0.349	0.253	**0.752**				
Geo_MSLQ	0.148	0.768	0.318	0.341	0.909	**0.671**			
Geo_Task	0.057	0.259	0.506	0.644	0.233	0.186	**0.673**		
Geo_Prob	0.234	0.467	0.331	1.172	0.311	0.404	0.281	**0.605**	
Alge_Prob	0.002	0.229	0.235	0.967	0.202	0.268	0.267	0.991	**0.613**

Alge_Task, Number and Algebra part in General-Math-Task-referenced self-efficacy questionnaire; Prac_MSLQ, Synthesis and Practice part in Motivated Strategies for Learning Questionnaire; Geo_MSLQ, Graphics and Geometry part in Motivated Strategies for Learning Questionnaire.For example, 0.590 represents the correlation of questions between “Number and Algebra” in General-Math-Task-referenced self-efficacy questionnaire, and other bold numbers represent the correlation of questions between trait in a certain method.

SRMR stands for “standardized root mean square residual,” whose value is between 0.0 and 1.0. The value obtained by a well-fitted model is less than 0.05, but a value up to 0.08 is considered good enough (Hooper et al., 2008). The indicators of all models in Table 4 were considered to be around 0.08, which was acceptable.

### 3.3. Discriminant validity of each factor of the model

Pohl and Steyer (2012) proposed that if the analyzed trait can be distinguished from other traits, the discriminant validity is supported. If different measurement methods produce similar results when measuring the same trait, the convergent validity can be proven.

Table 5 shows the correlation coefficient of CFA in model 4. For example, the correlation coefficient of the Number and Algebra part in General-Math-Task-referenced self-efficacy was 0.590, which was above heterotrait-heteromethod (HTHM), heterotrait-homomethod (HThM), and homotrait-heteromethod (hTHM; Kenny, 2012) coefficients in the same row, indicating that the discriminant validity is good. The correlation coefficient of the Graphics and Geometry part in General-Math-Task-referenced self-efficacy was 0.673, which was higher than the coefficient of the same row or the same column, indicating that the discriminant validity is fine. The correlation coefficient of the Synthesis and Practice part in General-Math-Task-referenced self-efficacy was 0.759, which was higher than the coefficient of the same row or the same column, indicating that the discriminant validity is well.

The correlation coefficient of the Number and Algebra part in Unconventional-Math-Problem-referenced self-efficacy was 0.613, which was higher than the coefficient of the same row or the same column, indicating that the discriminant validity was average. The correlation coefficient of the Graphics and Geometry part in Unconventional-Math-Problem-referenced self-efficacy was 0.605, which was higher than the coefficient of the same row or the same column, indicating that the discriminant validity was general. The correlation coefficient of the Synthesis and Practice part in Unconventional-Math-Problem-referenced self-efficacy was 0.637, which was higher than the coefficient of the same row or the same column, indicating that the discriminant validity is general.

The correlation coefficient of the Number and Algebra part in MSLQ was 0.752, which was higher than the coefficient of the same row or the same column, indicating that the discriminant validity is general. The correlation coefficient of the Graphics and Geometry part in MSLQ was 0.671, which was higher than the coefficient of the same row or the same column, indicating that the discriminant validity is general. The correlation coefficient of the Synthesis and Practice part in MSLQ was 0.682, which is higher than the coefficient of the same row or the same column, indicating that the discriminant validity is general. In conclusion, the discriminant validity of the first-order model of CFA was acceptable.

**Figure 1:**
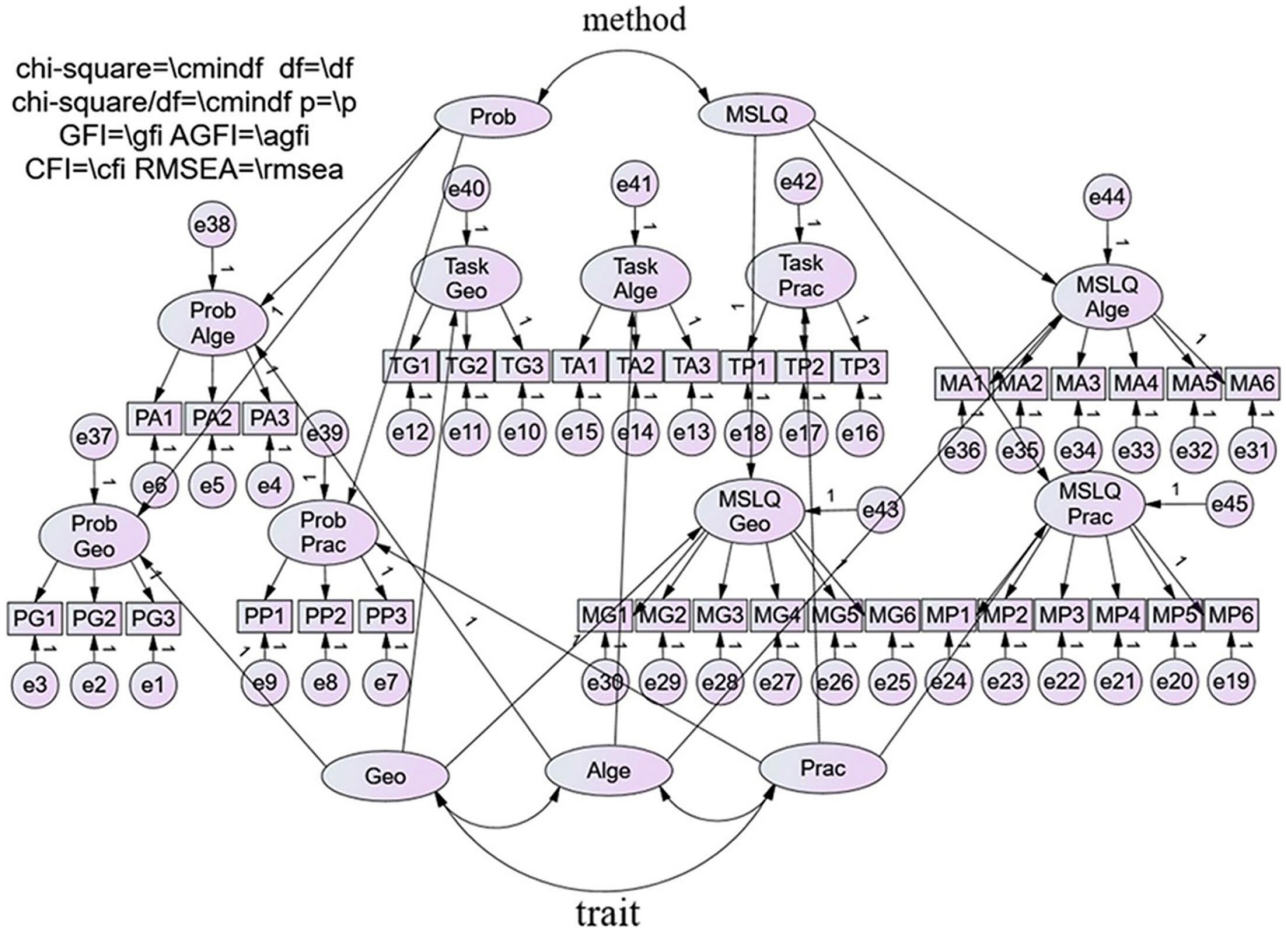
High-order confirmatory factor analysis model of multitrait-multimethod data with three correlated traits and two correlated method second-order factors with no trait–method correlation (model D). Prob, Unconventional-Math-Problem-referenced self-efficacy; MSLQ, Motivated Strategies for Learning Questionnaire; Geo, Graphics and Geometry self-efficacy; Alge, Number and Algebra self-efficacy; Prac, Synthesis and Practice self-efficacy.

To sum up, according to the research issue, we can measure individuals’ mathematics self-efficacy by using MTMM and get the most appropriate model, as shown in Figure 1.

### 3.4. Discussion

This research found that individuals’ mathematics self-efficacy can be measured by using MTMM and by constructing a high-order CFA model. In fact, certain psychological characteristics can be measured with MTMMs. Wang (2015) suggested using various ways of assessing and evaluating the multiple facets of metacognition and MTMM. Many researchers use the MTMM method as a measurement of psychological characteristics. Campbell et al. (2019) provided one example that considered using validating observation to comprehend how people teach in the university, with the use of modeling MTMM. Donaldson et al. (2020) examined two positive psychology structures using an MTMM research design. These studies had proved that MTMM is a professional psychological measurement method. Hidden variable models like CFA models and SEMs were proposed before 1990, to analyze the process of variability (Koch et al., 2017). Researchers do not agree on which parameterization of the CFA model characterizes MTMM data the most (Conway et al., 2004). At present, many studies support using CFA for MTTM figures. Conway et al. (2004) proposed that CFA supplies a most inclusive way of assessing MTMM figures. Nimlyat et al. (2018) proposed that other ways of assessing matrices of MTMM clearly favor using CFA. Kamakura and Ozer (2007) proposed that common matrixes in MTMM contain correlations between various traits calculated in various ways and let people assess how similar the ways are, i.e., convergence, and how unique they are, i.e., discrimination. A total of eight models were constructed in this study, which supported and verified the discriminant validity in the model of first-order CFA and the convergent validity of each facet. As indicated earlier, the present research attempts to find out whether MTMM data can measure mathematics self-efficacy by constructing a model of high-order CFA, so the study constructed the first-order CFA model and the second-order CFA model to measure mathematics self-efficacy, which indicated that CFA can support MTMM data for data modeling. The MTMM method provides a new idea and method for measuring mathematics self-efficacy.

According to the results, it was found that “Synthesis and Practice” had the most significant impact among the three traits, and MSLQ had the biggest impact on those three methods. “Synthesis and Practice” is a comprehensive field generated based on “Number and Algebra” and “Graphics and Geometry.” The field of “Synthesis and Practice” applied the learned mathematics knowledge to specific life practices, which emphasize that mathematics knowledge originates from life and is applied to life, and helps students better understand knowledge at a certain level. In the present study, MSLQ is used to measure students’ recognition degree of “Number and Algebra,” “Graphics and Geometry,” and “Synthesis and Practice,” which is about academic events of mathematics knowledge. If the questionnaire is applied to specific classroom learning, it can give feedback on students’ achievements in time, help students improve their achievements, and improve students’ mathematical self-efficacy level.

Bong and Hocevar (2002) used two methods and three traits to measure self-efficacy by constructing models, which verified the effectiveness of the discriminant validity and convergent validity of the model of higher order CFA. This study used two methods and three traits to construct a high-order CFA model specifically for measuring math learning self-efficacy. When verifying the goodness-of-fit data of the second-order CFA model, it was found that the goodness-of-fit figures for those two methods of Unconventional-Math-Problem-referenced self-efficacy and MSLQ self-efficacy, and three traits were better in the second-order model, that is, the correlation between the Unconventional-Math-Problem-referenced self-efficacy and MSLQ is higher than that of General-Math-Task-referenced self-efficacy. Unconventional-Math-Problem-referenced self-efficacy involves problem-posing and problem-solving, including asking students to pose problems of varying levels and solve problems. Future research can use Unconventional-Math-Problem-referenced self-efficacy and MSLQ, which can use the MTMM method to measure students’ level of mathematics self-efficacy by constructing models. According to the evaluation results, educators could let students raise their self-efficacy in math through intervention in different areas of mathematics. The future research objects can be concentrated in the field of primary and secondary schools, but should not be limited to school age only. Objects with psychological traits that need to be measured can be much more. Future research can expand the scope of traits or methods, not only limited to three traits and three methods. Current research is calculating mathematics self-efficacy using the characteristics of the subject. The measurement field of future research can be transformed into the self-efficacy of a certain subject or the self-efficacy of the STEM field, and the self-efficacy can be improved using answers from research.

There are still some limitations in this study. First, seventh-grade students are chosen as the participants, and the representatives of the sample need to be improved. Second, this study only shows the degree of influence of certain traits and methods on mathematics learning self-efficacy; whether and how to use the research results to improve students’ academic performance in the future needs further research.

## 4. Conclusion

This research draws the following conclusions. (1) MTMM can measure mathematics self-efficacy by establishing a high-order CFA model. (2) Through the screening method of MTMM, the “Synthesis and Practice” field of the three traits can better reflect students’ self-efficacy. (3) Through the screening method of MTMM, among the three methods, MSLQ can better reflect the self-efficacy of students.

## Data availability statement

The original contributions presented in the study are included in the article/Supplementary material, further inquiries can be directed to the corresponding author.

## Ethics statement

The studies involving human participants were reviewed and approved by the Ethics Committee of the Shandong Normal University. Written informed consent to participate in this study was provided by the participants’ legal guardian/next of kin.

## Author contributions

WY contributed to the basic conception and design of the study. WY and SZ wrote the first draft of the manuscript and wrote sections of the manuscript, committed to the organization of the questionnaire, and contributed to the construction of the specific structure. SZ contributed to the collection of original data, data analysis, and model construction. YZ contributed to the revision of the thesis grammar. WY and YZ put forward some professional suggestions on the specific contents and parts of the manuscript. All authors contributed to the manuscript revision and read and approved the submitted version.

## Funding

This research was supported by the Humanities and Social Sciences Project of the Chinese Ministry of Education, A Study on the Effectiveness of Individual Problem-Solving Interventions Based on BEA (No. 20YJAZH124), and Research Project of Humanities and Social Sciences in Colleges and Universities of Shandong Province, Exploration of Cognitive Mechanism of Mathematics Problem posing (No. J14WH07).

## Conflict of interest

The authors declare that the research was conducted in the absence of any commercial or financial relationships that could be construed as a potential conflict of interest.

## Publisher’s note

All claims expressed in this article are solely those of the authors and do not necessarily represent those of their affiliated organizations, or those of the publisher, the editors and the reviewers. Any product that may be evaluated in this article, or claim that may be made by its manufacturer, is not guaranteed or endorsed by the publisher.
