# Supplementary material for: Measuring mathematics self-efficacy: Multitrait-multimethod comparison
Source: Front Psychol. 2023 Mar 7;14:1108536. doi: 10.3389/fpsyg.2023.1108536 (PMC10028075; doi:10.3389/fpsyg.2023.1108536)

Supplementary Material

Measuring Math-Self-Efficacy: Multitrait-Multimethod Comparison

Wenhua Yu[1], Shuodi Zhou[1*], Yu zhou[2]

*** Correspondence:** Shuodi Zhou: Shuodzhou123@163.com

1 Supplementary Material：Questionnaires of Questions

Part I: Unconventional-Math-Problem-referenced self-efficacy Questionnaire

1. The beauty of the Four Leaf Diagram:

(1) In the square with side length of 4, draw 4 semicircles respectively with each side as the diameter to get a quadrangle as shown in Figure 1. Try to draw as many plane areas with different areas as possible by using shadows. In order to draw more plane areas with different areas, can you find some rules?

(2) Can you ask a question similar to (1) in Figure 2? What graphics do you think can be designed for research?

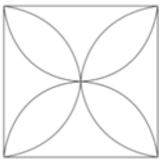

Figure 1

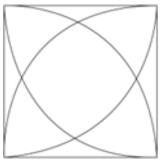

Figure 2

Q: On a scale of 0 to 7, how confident are you in your answer to this question?_______

2. Two numbers whose sum and product are equal:

What two numbers, their sum is equal to their product? You'll probably think of $2+2=2\times2$ right away. In fact, there are many such numbers, such as $3+3/2=3\times3/2$

(1) Can you write some of these two numbers? Can you find some rules?

(2) Can you ask some similar questions? Choose one of your questions to explore.

Q: On a scale of 0 to 7, how confident are you in your answer to this question? _______

3. Guess the number game.

There is a TV game called "Guess Idioms". The rule is that every two people in the game form a group. The host shows a sign with the idiom to one of the two people (A), but the other person (B) can't see the idiom on the sign. Now, let's ask Party A to tell the idiom on the sign of Party B with one sentence (this sentence can't contain words in the idiom) or one action, and ask Party B to guess the idiom according to Party A's words or actions, Now let's change the idiom in the game into two integers, and ask Party A to use a sentence or a formula, and a figure to tell Party B the two numbers (also, it is required that the same number as on the brand cannot appear) If you are A, how will you tell B about the following groups?

(1) -1 and 1;

(2) 1 and 2;

(3) 0 and 2.

Q: On a scale of 0 to 9, how confident are you in your answer to this question?________

4. Draw 3 straight lines on the plane. How many parts can the plane be divided into?

(1) Try to consider several possible situations?

(2) If we want to make the answer to this question unique, what conditions can be added?

Q: On a scale of 0 to 8, how confident are you in your answer to this question?________

5. Please use different methods to compare the sizes of 17/18 and 20/21.

Q: On a scale of 0 to 6, how confident are you in your answer to this question?________

6. There are 12 numbers on the clock face of the wall clock: 1, 2, 3, ..., 11, 12. Add a plus sign "+" or a minus sign "—" between the 12 numbers to get an equation, so that the result of this equation is 0.

(1) Have you ever seen this problem? (   )

A. Yes   B. No   C. I can't remember

(2) There are more than one hundred different solutions to this question. Write down

four different solutions you think of.

(3) Do you have any more ways to find more answers?

Q: On a scale of 0 to 8, how confident are you in your answer to this question?________

7. At 3 × 3. In the grid paper, try to divide the grid paper into two congruent polygons by connecting grid points, as shown in the following figure.

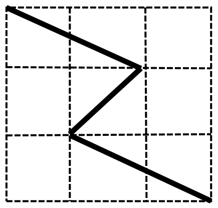

(1) Have you ever seen this problem? （ ）

A. Yes B. No C. I can't remember

(2) What other segmentation methods can you think of? Draw 5 different methods you think of in the figure below.

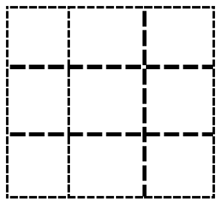

(3) What laws have you found that will help you find more answers?

Q: On a scale of 0 to 9, how confident are you in your answer to this question?________

8. Calculate and observe

$\begin{cases} 5\times5= \\ 4\times6= \end{cases}$ $\begin{cases} 8\times8= \\ 7\times9= \end{cases}$ $\begin{cases} 12\times12= \\ 11\times13= \end{cases}$ …….

According to the above mathematical situation, the teacher has put forward a mathematical question:

Known 25 × 25=625, then 24 × 26= ?

Please help the teacher continue to ask mathematical questions that meet the following requirements.

(1) Similar problems

(2) Other similar problems

Q: On a scale of 0 to 7, how confident are you in your answer to this question?_____

9. According to the formula “100/8=”, Compile 3 mathematical word problems related to real life. The answer to the first math word problem is 12, the answer to the second math word problem is 13, and the answer to the third math word problem is 12.5.

(1) Mathematical Application Problem 1:

(2) Mathematical Application Problem 2:

(3) Mathematical Application Problem 3:

Q: On a scale of 0 to 6, how confident are you in your answer to this question?_______

10. The following figure shows some cases where a straight line passes through a given square and exactly divides the square into two parts with equal area

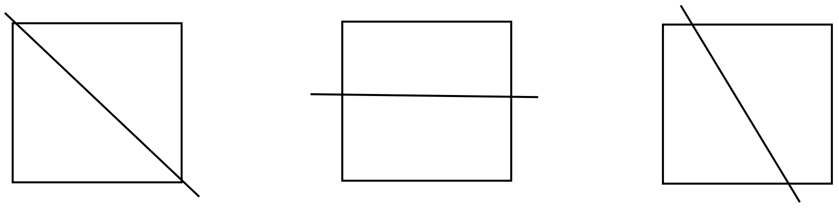

According to the above situations, ask mathematical questions that meet the following requirements.

(1) Easier mathematical problems:

(2) Mathematical problems to solve with some effort:

(3) A difficult mathematical problem:

Q: On a scale of 0 to 9, how confident are you in your answer to this question?_______

Note: Because question 9 is inconsistent with the cognitive level of the research object, this question will be deleted in the follow-up data analysis after discussion by experts.

Part II: Motivated Strategies for Learning Questionnaire

1. I'm certain that I can understand what is taught in a Number and Algebra class _____

A. Completely nonconforming B. Some nonconforming C. Slightly nonconforming

D. General E. Slightly conforming F. Some conforming G. Completely conforming

2. I expect to do very well in a Number and Algebra class _______

A. Completely nonconforming B. Some nonconforming C. Slightly nonconforming

D. General E. Slightly conforming F. Some conforming G. Completely conforming

3. I am sure that I can do an excellent job on the problems and tasks assigned for a Number and Algebra class _______

A. Completely nonconforming B. Some nonconforming C. Slightly nonconforming

D. General E. Slightly conforming F. Some conforming G. Completely conforming

4. I know that I will be able to learn the material for a Number and Algebra class _____

A. Completely nonconforming B. Some nonconforming C. Slightly nonconforming

D. General E. Slightly conforming F. Some conforming G. Completely conforming

5. My study skills are excellent in a Number and Algebra class _______

A. Completely nonconforming B. Some nonconforming C. Slightly nonconforming

D. General E. Slightly conforming F. Some conforming G. Completely conforming

6. I think I will receive a good grade in Number and Algebra class _______

A. Completely nonconforming B. Some nonconforming C. Slightly nonconforming

D. General E. Slightly conforming F. Some conforming G. Completely conforming

1. I’m certain that I can understand what is taught in a Graphics and Geometry class _____

A. Completely nonconforming B. Some nonconforming C. Slightly nonconforming

D. General E. Slightly conforming F. Some conforming G. Completely conforming

2. I expect to do very well in a Graphics and Geometry class _______

A. Completely nonconforming B. Some nonconforming C. Slightly nonconforming

D. General E. Slightly conforming F. Some conforming G. Completely conforming

3. I am sure that I can do an excellent job on the problems and tasks assigned for a Graphics and Geometry class _______

A. Completely nonconforming B. Some nonconforming C. Slightly nonconforming

D. General E. Slightly conforming F. Some conforming G. Completely conforming

4. I know that I will be able to learn the material for a Graphics and Geometry class_____

A. Completely nonconforming B. Some nonconforming C. Slightly nonconforming

D. General E. Slightly conforming F. Some conforming G. Completely conforming

5. My study skills are excellent in a Graphics and Geometry class _______

A. Completely nonconforming B. Some nonconforming C. Slightly nonconforming

D. General E. Slightly conforming F. Some conforming G. Completely conforming

6. I think I will receive a good grade in Graphics and Geometry class _______

A. Completely nonconforming B. Some nonconforming C. Slightly nonconforming

D. General E. Slightly conforming F. Some conforming G. Completely conforming

1. I’m certain that I can understand what is taught in a Synthesis and Practice class_____

A. Completely nonconforming B. Some nonconforming C. Slightly nonconforming

D. General E. Slightly conforming F. Some conforming G. Completely conforming

2. I expect to do very well in a Synthesis and Practice class _______

A. Completely nonconforming B. Some nonconforming C. Slightly nonconforming

D. General E. Slightly conforming F. Some conforming G. Completely conforming

3. I am sure that I can do an excellent job on the problems and tasks assigned for a Synthesis and Practice class _______

A. Completely nonconforming B. Some nonconforming C. Slightly nonconforming

D. General E. Slightly conforming F. Some conforming G. Completely conforming

4. I know that I will be able to learn the material for a Synthesis and Practice class_____

A. Completely nonconforming B. Some nonconforming C. Slightly nonconforming

D. General E. Slightly conforming F. Some conforming G. Completely conforming

5. My study skills are excellent in a Synthesis and Practice class _______

A. Completely nonconforming B. Some nonconforming C. Slightly nonconforming

D. General E. Slightly conforming F. Some conforming G. Completely conforming

6. I think I will receive a good grade in Synthesis and Practice class _______

A. Completely nonconforming B. Some nonconforming C. Slightly nonconforming

D. General E. Slightly conforming F. Some conforming G. Completely conforming

Part III: General-Math-Task-referenced self-efficacy Questionnaire

1. Simplify then evaluate: 2 (a^2b -3ab) -3 (ab-2ab^2-2), where a= - 2, b=1/2.

Q: On a scale of 0 to 5, how confident are you in your answer to this question? _______

2. From the first place to the second place, the bus had to travel for 7 hours. After the expressway was opened, the average speed of the bus increased by 20 kilometers per hour, and it only took 5 hours to get there, Seek the distance between A and B.

Q: On a scale of 0 to 5, how confident are you in your answer to this question? _______

3. In the Nine Chapters of Arithmetic, there is a statement about "the art of surplus and deficiency". The original text is as follows:

Today, there are people who buy things together, eight for three; Seven is less than four. How many people are there? What are the prices?

The translation is: At present, some people buy an item together, each of them pays 8 yuan, with a surplus of 3 yuan. So if each person pays 7 yuan, you're still $4 short. How many people are there in total? What's the price of this item?

Please answer the above questions

Q: On a scale of 0 to 5, how confident are you in your answer to this question? _______

4. As shown in the figure, it is a geometry made of 10 identical small cubes

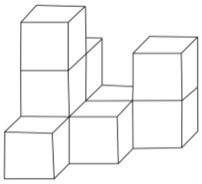

(1) Please draw its three views in the blank box.

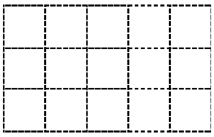

front view

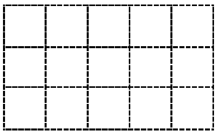

left view

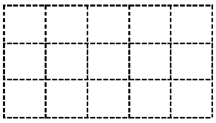

vertical view

(2) If the front view and vertical view remain unchanged, you can build up to_____ small cubes.

Q: On a scale of 0 to 6, how confident are you in your answer to this question? _____

5. As shown in the figure, it is known that O is a point on the straight-line AC, OB is a ray, OD bisects Angle AOB, OE is in Angle BOC, Angle BOE = 1/2 Angle EOC, DOE = 70°. Calculate the degree of Angle EOC.

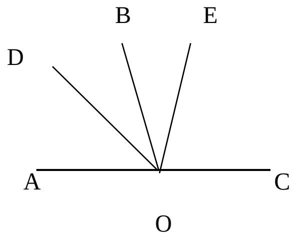

Q: On a scale of 0 to 8, how confident are you in your answer to this question?________

6. As shown in the figure, B and C divide line segment AD into three parts: 2:5:3. M is the midpoint of AD, BM= 6cm, and calculate the length of CM and AD.

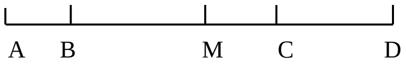

Q: On a scale of 0 to 6, how confident are you in your answer to this question?_______

7. During the New Year's Day of 2019, a mall will display promotional advertisements, as shown in the following Table:

Preferential conditions	No more than 200 yuan for one-time purchase	A one-time purchase of more than 200 yuan, but not more than 500 yuan	One time shopping more than 500 yuan
Discount	No discount	10% off for all	10% off for 500 yuan and 20% off for more than 500 yuan

(1) Expressed by algebraic expression (the result should be simplified)

Suppose the original price of a one-time purchase item is x yuan. When the original price of x exceeds 200 yuan but does not exceed 500 yuan. the actual payment is_______

yuan; When the original price exceeds 500 yuan, the actual payment is _______yuan;

(2) If A makes a one-time payment of 490 yuan, what is the original price of the item?

(3) If B makes two purchases, and the sum of the original prices of the two purchases is 1000 yuan (the original price of the second purchase is higher than that of the first purchase), and the actual payment for the two purchases is 894 yuan, what is the original price of the two purchases respectively?

Q: On a scale of 0 to 10, how confident are you in your answer to this question?________

8. The tons of cement imported into a cement warehouse within 7 days in a week are as follows (“+ ”means incoming and “ - ”means outgoing): +30, -25, -30, +28, -29, -16, -15.

(1) After 7 days, did the cement in the warehouse increase or decrease? How many tons have been increased or decreased?

(2) After seven days, the warehouse manager found 200 tons of cement in the warehouse. Then how many tons of cement were in the warehouse 7 days ago?

(3) If the loading charge of cement into the warehouse is 15 yuan per ton, and the loading charge of cement out of the warehouse is 18 yuan, how many yuan should be paid in these 7 days

Q: On a scale of 0 to 9, how confident are you in your answer to this question? _______

9. A school in order to understand the school's seventh grade students' extracurricular reading preferences, randomly selected the seventh-grade students of the school to conduct a questionnaire survey (each student only selected one kind of book). The following is two incomplete statistical charts drawn after data collation. Please answer the following questions according to the information provided in the charts:

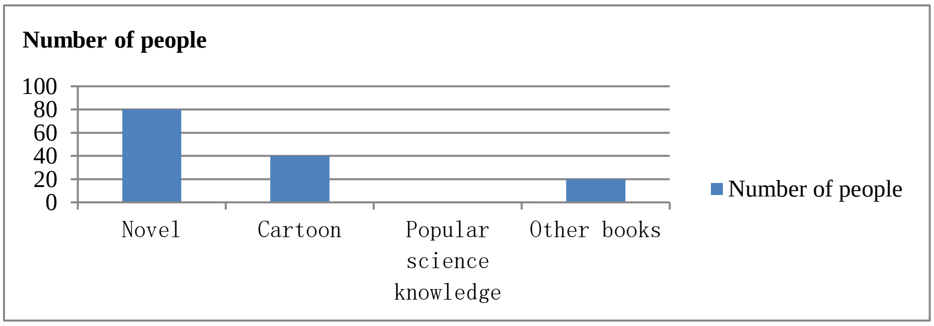

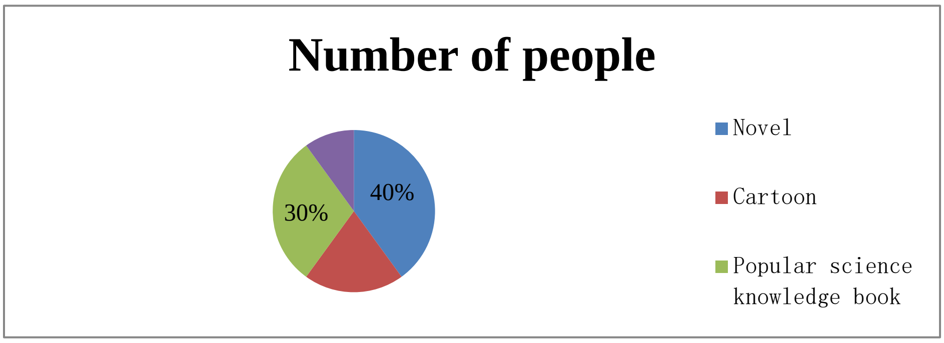

(1) A total of ______ students were investigated in this activity;

(2) In the sector statistical chart, the central Angle of the sector where "other" is ________located is equal to degrees;

(3) Complete bar statistical chart;

(4) If there are 600 students in this grade, what is your estimate of the number of students who like "popular science knowledge"?

Q: On a scale of 0 to 6, how confident are you in your answer to this question?________

2 Supplementary Figures and Tables

2.1 Supplementary Figures

Supplementary Figure 1. The Four models Figures for first-order confirmatory factor analysis include model 1, model 2, model 3, and model 4.

Model 1

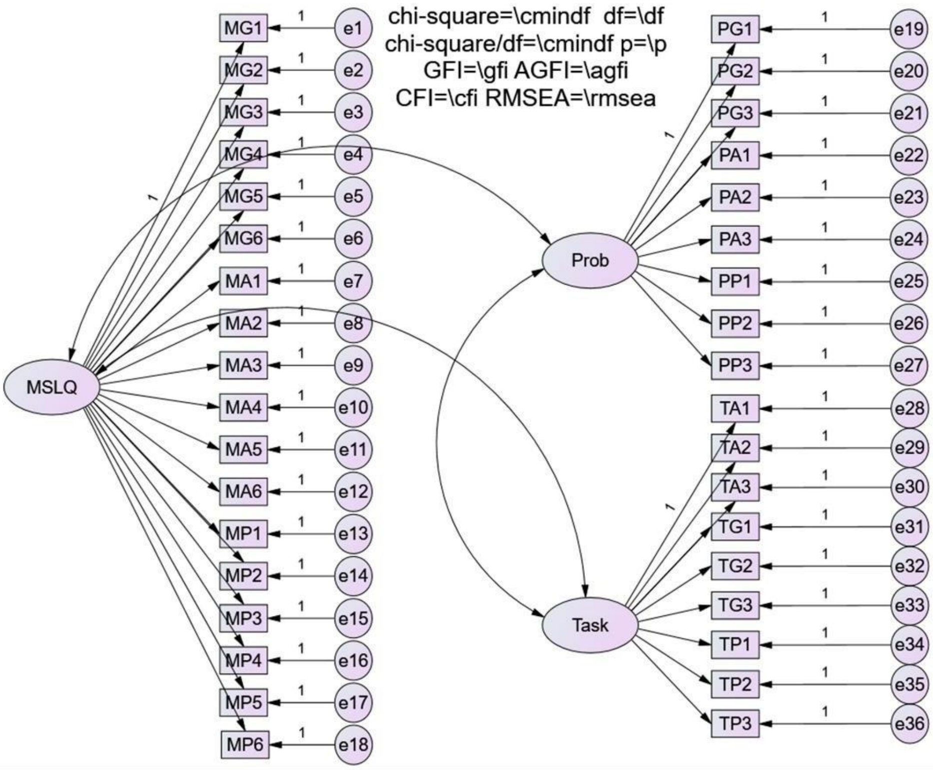

Model 2

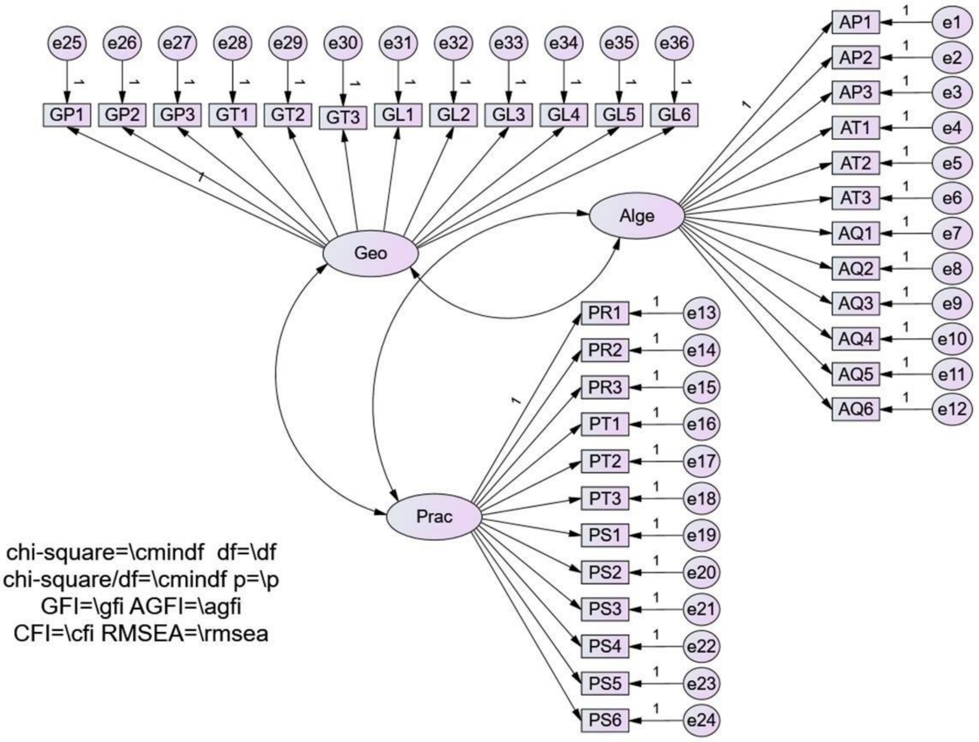

Model 3

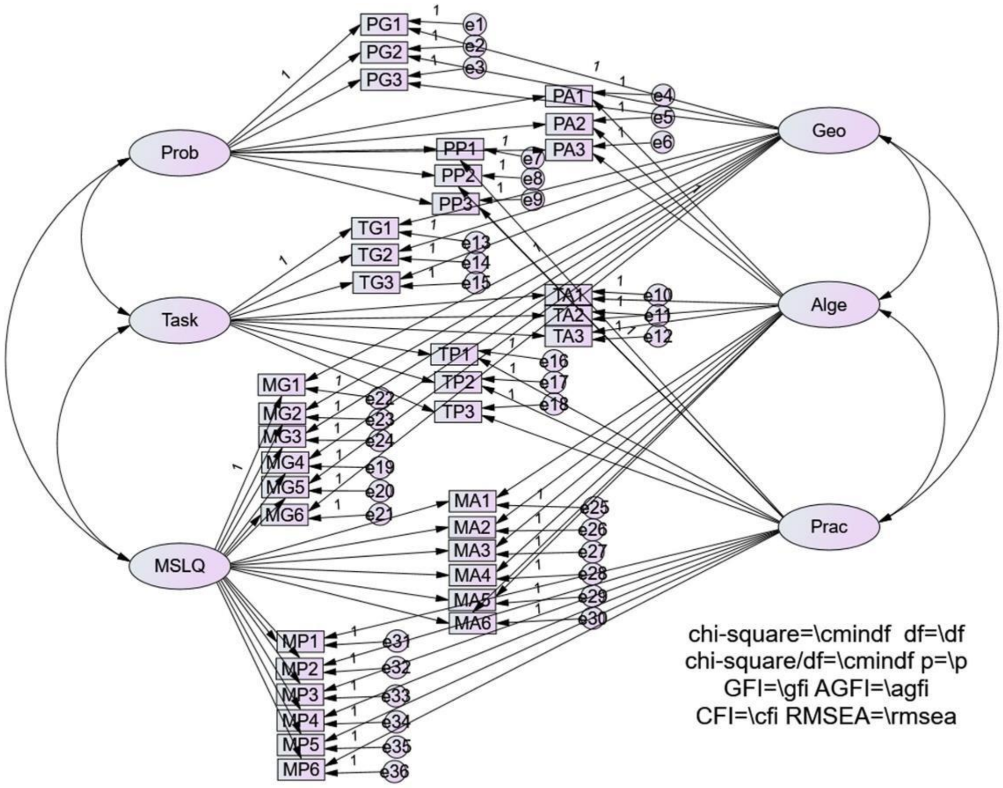

Model 4

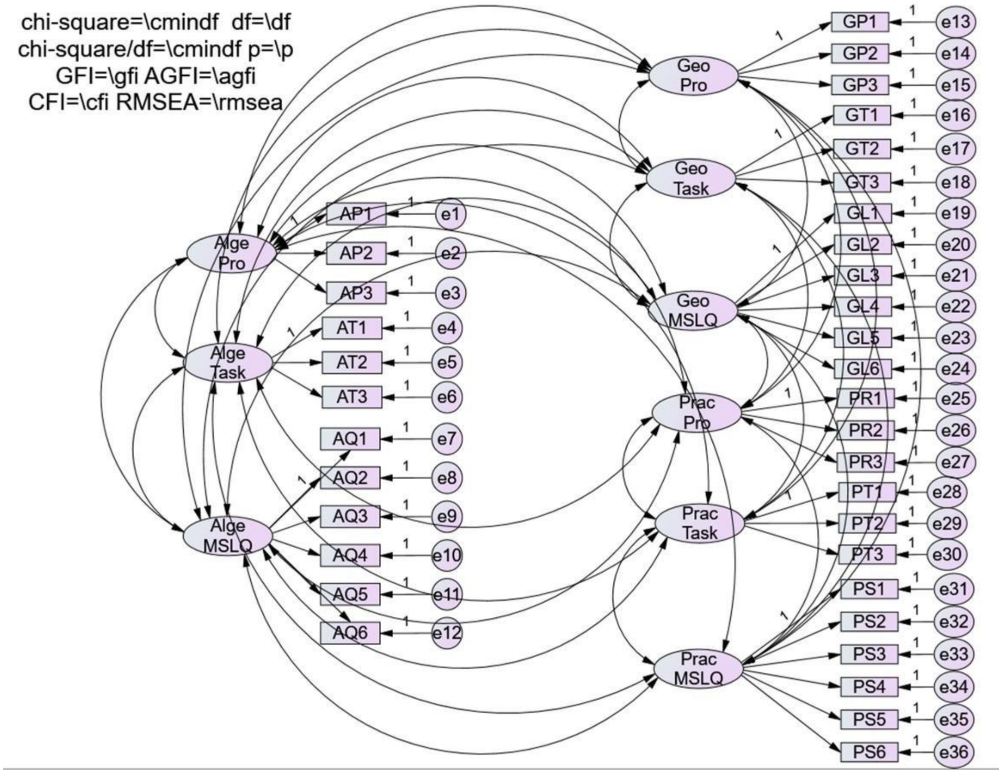

Supplementary Figure 2. The Four models Figures for second-order confirmatory factor analysis include model A, model B, model C, and model D.

Model A

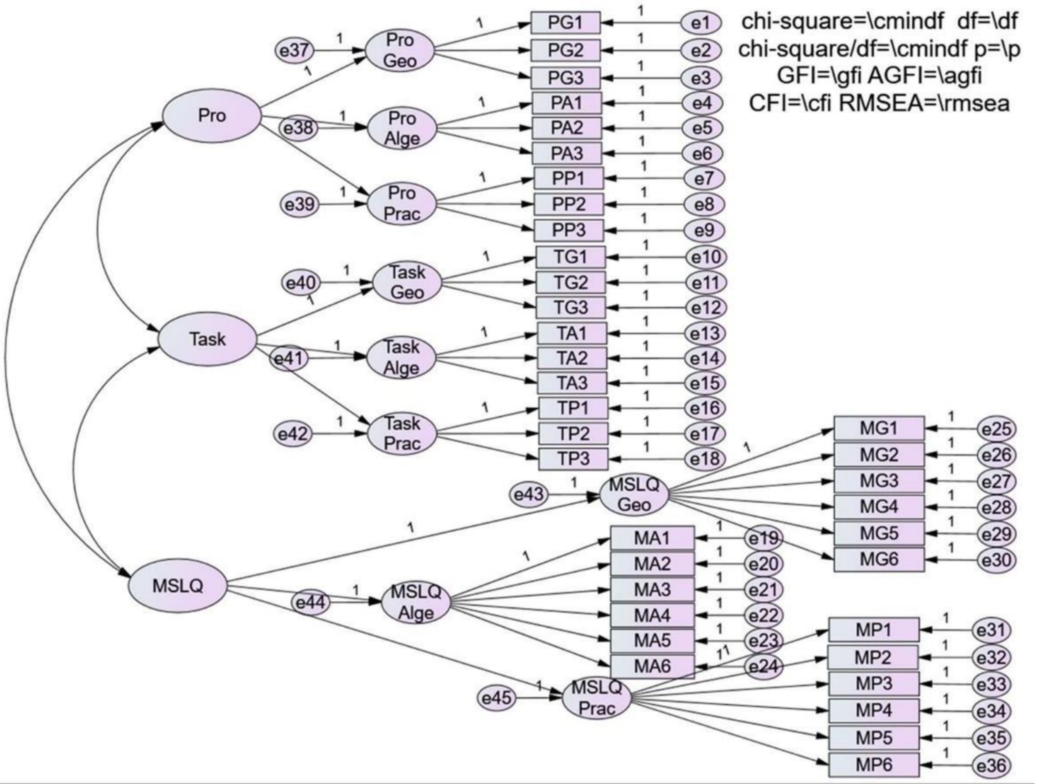

Model B

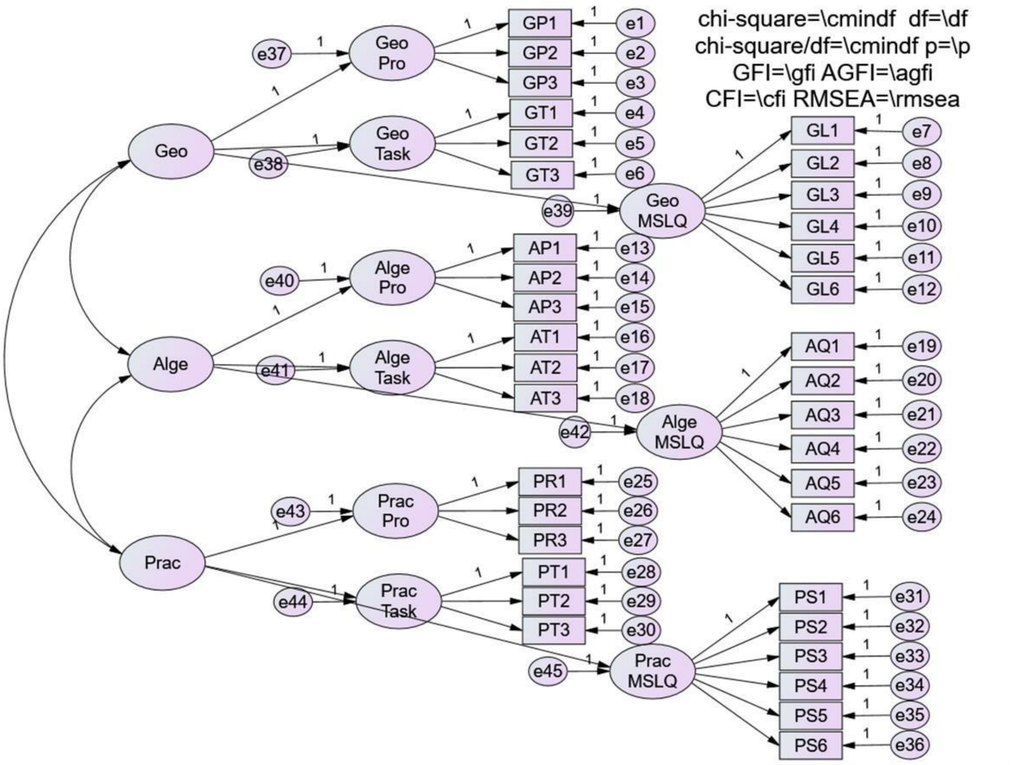

Model C

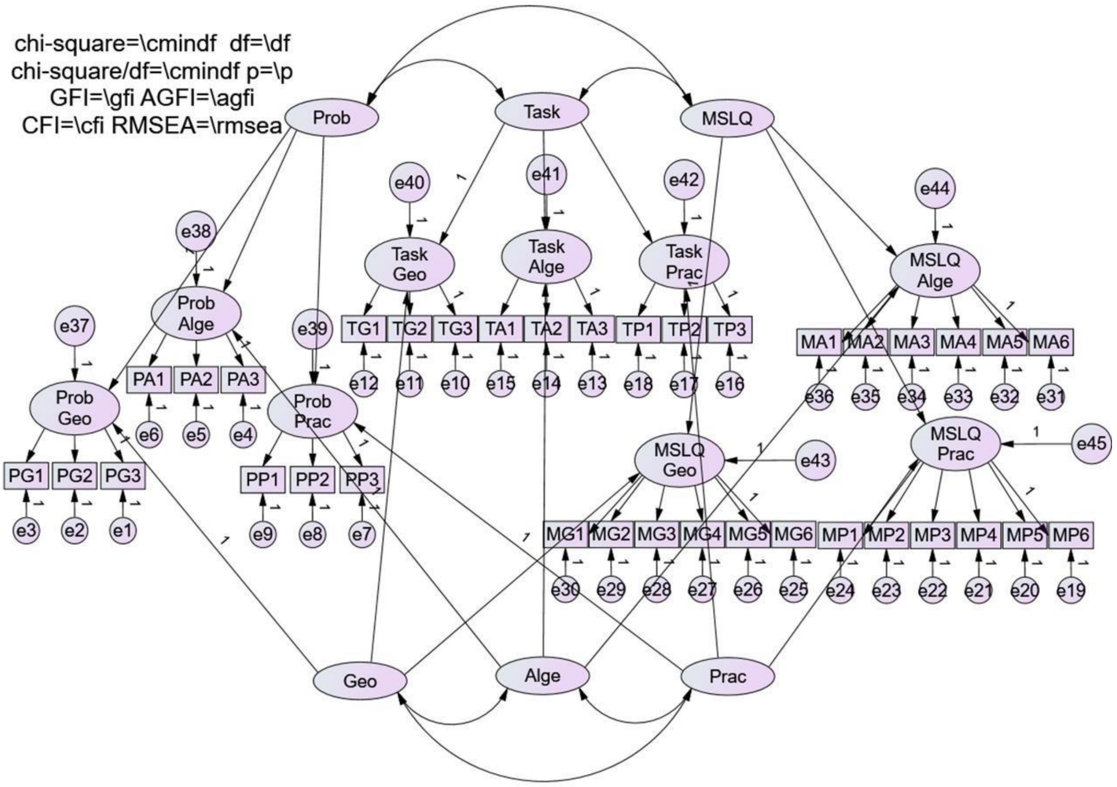

Model D

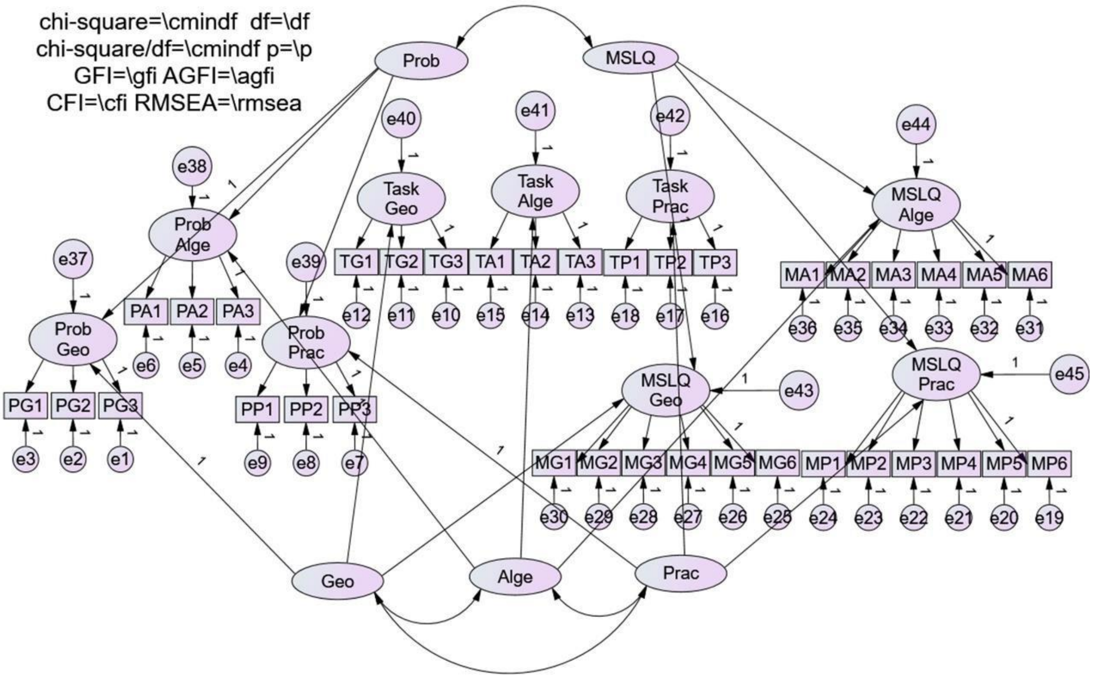

Supplement: Supplementary file 1 [file Data_Sheet_1.pdf]
